# Sacroiliitis and Polyarteritis Nodosa in a Patient with Familial Mediterranean Fever

**DOI:** 10.1155/2016/5134546

**Published:** 2016-04-07

**Authors:** Yunus Ugan, Atalay Doğru, Hüseyin Şencan, Mehmet Şahin, Şevket Ercan Tunç

**Affiliations:** ^1^Faculty of Medicine, Department of Internal Medicine, Division of Rheumatology, Suleyman Demirel University, 32260 Isparta, Turkey; ^2^Faculty of Medicine, Department of Internal Medicine, Suleyman Demirel University, 32260 Isparta, Turkey

## Abstract

Familial Mediterranean fever (FMF) is an autoinflammatory disorder with autosomal recessive inheritance, characterized by recurrent fever and episodes of serositis. The condition is known to be caused by mutations in the MEFV (Mediterranean FeVer) gene, located in the short arm of chromosome 16. While more than 310 sequence variants in the MEFV gene have been described to date, the diagnosis is still established clinically. FMF may be accompanied by sacroiliitis and various forms of vasculitis. The most common forms of associated vasculitis are Henoch-Schonlein purpura and polyarteritis nodosa (PAN). We have presented here a fairly rare case of FMF, accompanied by both sacroiliitis and PAN.

## 1. Introduction

Familial Mediterranean fever (FMF) is an autoinflammatory disorder, which commonly manifests as fever characterized by episodes of serositis involving the peritoneum, pleura, synovium, and rarely the pericardium. If no treatments are taken, FMF may lead to secondary amyloidosis; thus it is associated with high mortality and morbidity [1].

Arthritis is a very common finding in FMF, typically nonmigratory monoarthritis localized in the joints of lower extremities, without any sequelae. In very rare instances it may cause chronic arthritis or sacroiliitis that may lead to damage in the hip joint [2].

Seven percent of the patients with FMF may have Henoch-Schonlein purpura (HSP) and 1% may have polyarteritis nodosa (PAN) [3]. When compared to the general population, this incidence rate is very high. Case studies of FMF accompanied by either spondyloarthropathy (SpA) group of disorders or PAN have been reported in the literature; however, cases where these three disorders occur concomitantly have not been reported. The following case therefore reports a rare case of PAN and sacroiliitis in a patient with FMF.

## 2. Case Presentation

**Figure 1:**
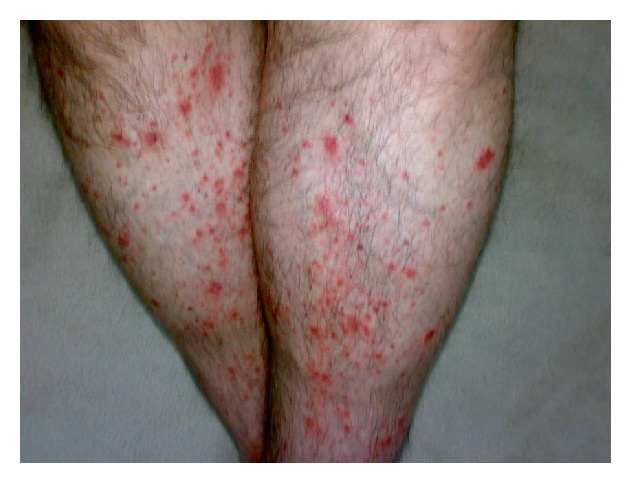
Bilateral palpable purpura in lower extremities.

**Figure 2:**
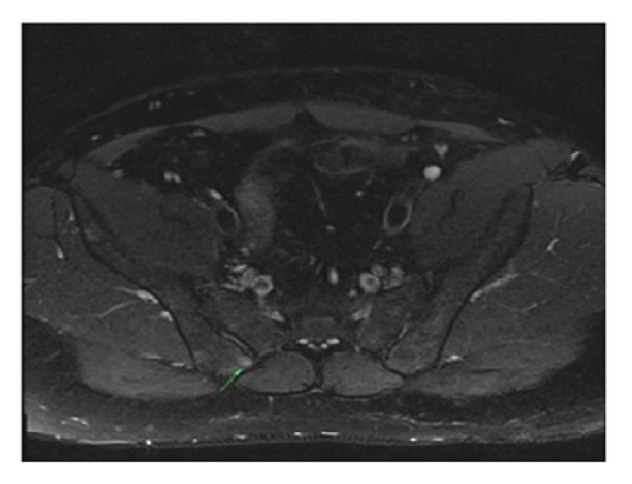
Sacroiliac joint magnetic resonance imaging was consistent with sacroiliitis at right posterior of the sacroiliac joints.

A 22-year-old male presented with complaints of abdominal pain, joint pain, fever, and rash. The occurrence of abdominal pain was described as occasional, starting from when the patient was about 5 years old. Each episode was reported to last for about 3 days. The pain started specifically from the epigastric region, then spread to the whole abdomen, and was accompanied by high temperature of about 39°C. The patient also experienced asymmetrical pain, particularly in the ankles, the knee, the hip joint, and elbows. Starting from 6 months prior to the current study, the patient suffered from back pain, which occurred particularly after resting and was accompanied by 1 hour of morning stiffness. Nonitchy skin rash with swelling and redness in the lower extremities started a week prior to the current study (Figure 1). The patient also had a palpable hardness of 2 × 3 cm size in the posterior left foot, cruris region below the knee, which began 10 days previously. The patient described excessive muscular pain and 8 kg weight loss in the last 3 months. The patients' family history included a cousin with a diagnosis of FMF. The physical examination results were as follows: pulse: 120/min, BP: 160/95 mmHg, and body temperature: 38.5°C. The patient had no rebound tenderness but mild defense in the abdomen and red swollen petechial skin lesions in the extremities. Laboratory tests indicated the following parameters: Hb: 13 g/dL, WBC: 17,000/*μ*L, platelet: 330,000/mm^3^, erythrocyte sedimentation rate (ESR): 52 mm/h, C-reactive protein (CRP): 35 mg/dL (N: 0–3), creatinine: 1.5 mg/dL (N: 0.6–1.2), ferritin: 114 ng/dL (N: 14–150), HBsAg (+), anti-HBs (−), HBeAg (−), anti-HBe (+), and HBV-DNA: 3.1 × 10^3^ IU/mL. Occult blood in stool, urinary microscopy, brucella, anti-HCV, rheumatoid factor, HLA B27, anti-nuclear antibody (ANA), anti-neutrophilic cytoplasmic antibody (ANCA), and extractable nuclear antigens panel were negative. There was no growth in the blood and urine cultures. An FMF gene mutation (M694V homozygous positive) was identified. The presence of sacroiliitis was established by sacroiliac joint magnetic resonance imaging (SIJ MRI) (Figure 2). Superficial ultrasonography performed to investigate the palpable hardness in the cruris region showed consistency with intramuscular hematoma. Renal Doppler and renal arteriography were performed to investigate potential PAN due to the presence of petechia-purpura, myalgia, positive HBsAg, and hypertension; however, no pathology was revealed. Echocardiography showed cardiomyopathy secondary to hypertension. Abdominal ultrasonography revealed no pathology. Muscular and fat tissue biopsies were performed. While no prominent pathology was detected in the skin and subcutaneous fatty tissue, lymphocyte, leukocyte, plasma cell, and eosinophil infiltrations were observed around the medium- and small-diameter vessels in the muscle tissue. Cellular infiltration had destroyed vascular walls and caused obstructions in medium-diameter vessels due to fibrin accumulation. Based on available findings and consistent clinical characteristics, the patient was diagnosed with FMF. The patient was initiated on colchicine 1 mg/day due to the high creatinine level. Concurrent presence of sacroiliitis with FMF was considered; however, nonsteroidal anti-inflammatory medication was not given due to the presence of renal failure; instead sulfasalazine 2000 mg/day was initiated. The presence of rash and hypertension, high level of creatinine, positive hepatitis B serology, cardiomyopathy secondary to hypertension, presence of muscular pain, and concomitant weight loss suggested a diagnosis of PAN. Accordingly, a moderate dose of corticosteroid and azathioprine was added to his treatment; however, he was started on lamivudine (100 mg/day) due to the positive hepatitis B serology before immunosuppressive therapy. ESH and CRP values returned to normal ranges in the 1st month of treatment; the patient started gaining weight and had a complete resolution of joint pain.

## 3. Discussion

The case presented here demonstrated clinical and laboratory features that were consistent with FMF. The patient was also homozygous positive for the M964V gene mutation. A concomitant back and hip pain together with morning stiffness primarily suggested sacroiliitis. Moreover, SIJ MRI revealed the presence of sacroiliitis. Langevitz et al. reported an incidence of concomitant SpA in 0.4% of FMF patients who were usually HLA-B27 negative, although the exact values remain unknown [4]. In a study by Akar et al. including 201 patients with FMF, the frequency of AS was 7.5% and axial SpA was 8.9% [5]. Kasifoglu et al. similarly reported that the frequency of sacroiliitis in FMF was found to be 7% [6]. Compared to other SpAs, the clinical manifestations of patients with concurrent FMF do not include uveitis, involvement of anterior chest wall, or significant changes in spinal radiology [7]. Previous publications have suggested that mutations of the MEFV gene were involved in the pathogenesis of patients with HLA-B27 negative ankylosing spondylitis (AS). However, Maraş et al. showed that the mutations in the MEFV gene were not increased in patients with AS compared to the normal population showing a lack of clinical involvement of the gene product [8].

FMF patients are known to have a higher prevalence of vasculitis such as PAN and HSP compared to the overall population [3]. Patients who develop PAN in the background of FMF exhibit a younger age of onset of the disease [9]. Classical manifestations of PAN include fatigue, fever, rash, muscular pain, joint pain, abdominal pain, and hypertension. Since an increased acute phase response occurs in both disorders, the diagnosis of PAN becomes difficult in FMF patients due to similar clinical and laboratory findings. Angiographic examinations or invasive methods such as biopsy may be required to establish the diagnosis. Since the patient in the current study had a normal angiography along with clinical findings such as widespread muscular pain, weight loss, positive HBsAg, hypertension, and high levels of creatinine, all of which supported the diagnosis of PAN, a decision for skin and muscle biopsy was made. The results of the biopsy were consistent with PAN in medium- and small-diameter vessels.

Diseases such adult Still's disease (normal ferritin, absence of sore throat and organomegaly, and inconsistent rash), brucella (brucella tube agglutination negative), ANCA-associated vasculitis (ANCA negative, no pulmonary involvement, normal urinary sediment, and no asthma history), systemic lupus erythematosus (ANA negative, anti-ds-DNA negative, normal complement level, and no hematologic or renal involvement) were considered in the differential diagnosis, and relevant investigations were conducted.

Mild cases of PAN and cases localized only to the skin are treated with glucocorticoids, while cyclophosphamide in combination with glucocorticoids is used in moderate-to-severe cases for the induction of remission. Methotrexate, azathioprine, or mycophenolate mofetil are used in maintenance treatment. While there are no randomized controlled studies on TNF alpha blockers and rituximab in refractory patients, there are publications reporting good responses to treatment [10, 11]. In cases of hepatitis B-associated PAN, antiviral treatment and plasmapheresis are more commonly used in comparison to immunosuppressive agents [12]. These patients should definitely be administered antiviral therapy before treatment. Since the patient in the current study had mild-to-moderate disease, he was given immunosuppressive treatment with antiviral therapy without plasmapheresis, and a positive response was achieved.

## 4. Conclusion

During the follow-up of FMF patients, in addition to amyloidosis, concomitant sacroiliitis and vasculitis should be considered, and relevant symptoms should be thoroughly investigated.
